# Case report: Toceranib as adjuvant chemotherapy in a dog with incompletely resected combined hepatocellular-cholangiocarcinoma

**DOI:** 10.3389/fvets.2022.963390

**Published:** 2023-01-04

**Authors:** Sang-Won Kim, Ju-Won Choi, Jeon-Mo Kim, Hun-Young Yoon, Kieun Bae, Kyong-Ah Yoon, Jung-Hyun Kim

**Affiliations:** ^1^KU Animal Cancer Center, Konkuk University Veterinary Medical Teaching Hospital, Seoul, South Korea; ^2^Department of Veterinary Internal Medicine, College of Veterinary Medicine, Konkuk University, Seoul, South Korea; ^3^Department of Veterinary Surgery, College of Veterinary Medicine, Konkuk University, Seoul, South Korea; ^4^Department of Veterinary Biochemistry, College of Veterinary Medicine, Konkuk University, Seoul, South Korea

**Keywords:** chemotherapy, combined hepatocellular-cholangiocarcinoma, dog, toceranib, tyrosine kinase inhibitor

## Abstract

An 11-year-old intact female mixed breed dog was presented with abdominal distention and elevated hepatic enzyme levels. Computed tomography revealed a multicystic hepatic mass at the left medial lobe adjacent to the diaphragm and caudal vena cava. The mass was surgically removed with partial hepatectomy, but it could not be removed completely because of adhesion to the diaphragm. The tissue was submitted for histopathologic evaluation, and the patient was diagnosed with stage IIIA combined hepatocellular-cholangiocarcinoma (cHCC-CC). Considering the residual tumor tissue from incomplete surgical excision, adjuvant chemotherapy was recommended. Tumor tissue obtained from the patient was assessed using an anticancer drug response prediction test, and the results showed that toceranib phosphate was the most effective chemotherapeutic agent for this patient. Toceranib was initiated (3.1 mg/kg, PO, q48 h), and routine adverse effect assessment, including systemic blood pressure measurement, complete blood count, serum biochemical evaluations, and urinalysis were performed at two-week intervals for the first 2 months and every 2 months thereafter. Radiography and ultrasonography were conducted at one-month intervals for the first two months and then every 2 months subsequently. Concurrent hyperadrenocorticism was managed with trilostane (1 to 5 mg/kg, PO, q12h). The patient showed no critical adverse effects of chemotherapy, obvious recurrence, or metastasis. The response to toceranib was assessed as a partial response, and the patient is still alive over 23 months after tumor excision. This is the first case report describing chemotherapy for a dog with cHCC-CC.

## Introduction

Primary hepatobiliary tumors are uncommon, accounting for 0.6–2.6% of all canine tumors (1). There are four basic categories of primary malignant hepatobiliary tumors in dogs: hepatocellular carcinoma, bile duct carcinoma, neuroendocrine tumor (carcinoid), and sarcoma (2, 3). Hepatocellular carcinoma (HCC) is the most common malignant liver tumor in dogs followed by bile duct carcinoma, also known as cholangiocarcinoma (CC) (2, 4).

Combined hepatocellular-cholangiocarcinoma (cHCC-CC) is a rare primary hepatobiliary tumor in humans and dogs, with concurrent HCC and CC within the same neoplastic tissue (5, 6). The incidence rate of cHCC-CC in dogs has been reported to be 1.8 or 2.0% of all primary hepatobiliary tumors (3, 7).

All primary hepatobiliary tumors can be classified into massive, nodular, or diffuse types based on their gross morphology (3). Liver lobectomy is the treatment of choice for massive type hepatobiliary tumors (1, 4). However, nodular or diffuse type tumors are not amenable to surgical excision, and hence have a worse prognosis than that of the massive type (4). Therefore, other options, such as chemotherapy, may be considered for unresectable massive type, nodular, or diffuse type tumors (4).

Receptor tyrosine kinases (RTK) are normally expressed on the cell surface, and growth factors bind to these receptors to regulate their activation (8). In both human and veterinary medicine, tyrosine kinases have been identified to be abnormally activated in malignant tumor cells, inducing uncontrolled tumor cell proliferation and survival (8). Thus, tyrosine kinase inhibitors that block RTKs overexpressed in tumor cells can reduce the downstream signaling cascade and thereby exert antitumor activity.

Conventional chemotherapy agents are cytotoxic and induce indiscriminate cell death, but targeted agents kill tumor cells selectively, and therefore fewer side effects are expected (9). Toceranib phosphate is a tyrosine kinase inhibitor targeting vascular endothelial growth factor receptor (VEGFR), platelet-derived growth factor receptor (PDGFR), and c-KIT; thus, it shows both antiangiogenic and antitumor activities (8). Toceranib is an FDA-approved agent used in dogs with non-resectable Patnaik grade II or III mast cell tumors (10). Moreover, it has shown some efficacy in the treatment of many other spontaneous canine malignancies (10).

In this case report, a dog with cHCC-CC perforating the visceral peritoneum but without obvious regional lymph node or distant metastasis was treated with partial hepatectomy and adjuvant toceranib chemotherapy. Here, we describe the clinical manifestations and the diagnostic and therapeutic approach in this case and review the application of tyrosine kinase inhibitors in canine cHCC-CC.

## Case presentation

An 11-year-old intact female mixed breed dog was presented to Konkuk University Veterinary Medical Teaching Hospital (KU-VMTH) for investigation of elevated hepatic enzyme levels. The veterinarian at a local animal hospital initially evaluated and referred the dog to KU-VMTH without additional tests. The owner reported that the patient had recently started to show panting and polyphagia. Physical examination showed that vital signs, including body temperature, pulse rate, respiratory rate, and blood pressure were within normal range. However, abdominal distention and decreased muscle mass on the hindlimbs were palpable, and the patient was plantigrade on gait analysis. Generalized alopecia and suspected calcinosis cutis skin lesions were also observed.

Complete blood count (CBC) revealed thrombocytosis (784 × 10^3^/μL; reference range, 148–484 × 10^3^/μL), and serum biochemical assessments showed decreased creatinine (0.2 mg/dL; reference range, 0.5–1.8 mg/dL), elevated alanine aminotransferase (829 U/L; reference range, 10–100 U/L), aspartate aminotransferase (80 U/L; reference range, 0–50 U/L), alkaline phosphate (3,281 U/L; reference range, 23–212 U/L), and gamma-glutamyl transferase (247 U/L; reference range, 0–7 U/L) levels as well as hyperlactatemia (4.01 mmol/L; reference range, 0.5–2.5 mmol/L) and hypertriglyceridemia (214 mg/dL; reference range, 10–100 mg/dL). In addition, the coagulation profile showed a normal prothrombin time (PT), delayed activated partial thromboplastin time (aPTT) (>300 s; reference range, 72–102 s), normal D-dimer and elevated maximum amplitude (MA) (76.1 mm; reference range, 42.9–67.9 mm) on thromboelastography (TEG).

Based on the clinical signs and physical examination results, hyperadrenocorticism was strongly suspected and adrenocorticotropic hormone (ACTH) stimulation test was performed. The pre-ACTH cortisol (>10 μg/dL; reference range, 2–6 μg/dL) and post-ACTH cortisol (>30 μg/dL; reference range, 6–18 μg/dL) levels were both above the normal range. Moreover, urinalysis revealed an elevated urine protein to creatinine ratio (UPC) (3.61; reference range, <0.5).

**Figure 1:**
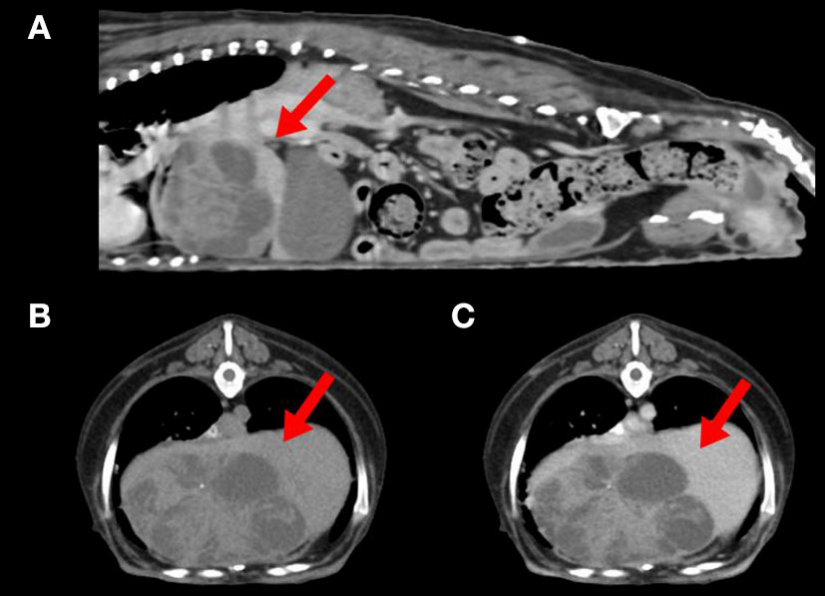
Computed tomography of a dog with combined hepatocellular-cholangiocarcinoma. **(A)** Sagittal view showing a left medial liver lobe multicystic mass (arrow) with adhesion to diaphragm and caudal vena cava. **(B)** Plain and **(C)** postcontrast axial view of the cranial abdominal region showing a hypoattenuated liver mass (arrows) with minimal contrast enhancement.

Abdominal radiographic examination showed hepatomegaly and resultant caudally deviated gastric axis. Abdominal ultrasonography showed a multicystic mass at the liver, which was suspected to originate from the left medial or right medial lobe. Computed tomography (CT) identified a left medial liver lobe multicystic mass measuring 7.4 × 4.8 × 4.9 cm, showing adhesions with the diaphragm, caudal vena cava, right medial lobe, and quadrate lobe (Figure 1). In addition, an enlarged pituitary gland and bilateral enlarged adrenal glands were detected. The abovementioned imaging modalities did not show evidence of metastasis to the thoracic or abdominal organs.

Pituitary-dependent hyperadrenocorticism was also provisionally diagnosed considering the CT scan results, although low-dose dexamethasone suppression test or high-dose dexamethasone suppression test results were not available.

Partial hepatectomy was performed to obtain a definitive diagnosis of the hepatic mass. As predicted, the hepatic mass was identified at the left medial lobe and showed considerable adhesion to the diaphragm, preventing complete excision. The mass was excised as much as possible but approximately 10% of the mass remained unresected. The excised hepatic mass was fixed in formalin and submitted to Antech Diagnostics for histopathologic evaluation.

**Figure 2:**
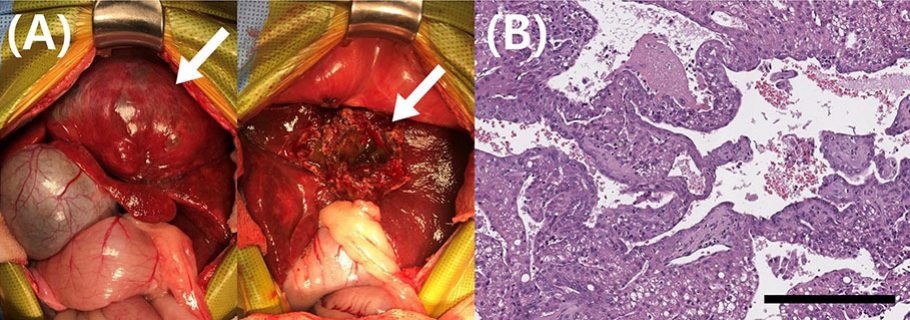
Gross morphological and histopathological findings of the liver mass in a dog with combined hepatocellular- cholangiocarcinoma. **(A)** During surgery, these pictures were taken before and after the partial hepatectomy. Complete excision of the liver mass was impossible because of significant adhesion to diaphragm. The mass (arrow) was excised as much as possible but about 10% of the mass (arrow) remained unresected. **(B)** Histopathology of the hepatic mass revealed that the hepatic parenchyma includes two overlapping proliferations. One section comprised tubules, cysts, and epithelial cell nests in fibrovascular stroma. The neoplastic cells had variably distinct cell borders, moderate amount of eosinophilic cytoplasm, and oval nuclei. Marked anisokaryosis and anisocytosis were observed with two mitotic figures in 10 high-power fields (400×). The second section comprised a hepatocellular neoplasm. Two to four hepatocyte-wide hepatic cords were observed, with loss of lobular architecture. Hepatocytes had abundant cytoplasm with central to peripheralized nuclei. Mild anisokaryosis and anisocytosis were observed with 12 mitotic figures in 10 high power fields (400×). Magnification 400 ×; scale bar = 50 μm. H&E, hematoxylin & eosin.

The pathologist reported that the hepatic parenchyma was partially replaced by a multinodular mass and that the lesion had two overlapping proliferations. One section comprised tubules, cysts, and epithelial cell nests in fibrovascular stroma. The neoplastic cells had variably distinct cell borders, moderate amount of eosinophilic cytoplasm, and oval nuclei. Marked anisokaryosis and anisocytosis were observed with two mitotic figures in 10 high-power fields (400×). The second section comprised a hepatocellular neoplasm. Two to four hepatocyte-wide hepatic cords were observed, with loss of lobular architecture. Hepatocytes had abundant cytoplasm with central to peripheralized nuclei. Mild anisokaryosis and anisocytosis were observed with 12 mitotic figures in 10 high power fields (400×). Immunohistochemical staining of hepatocellular cells for cytokeratin 7 (CK7) and hepatocyte paraffin 1 (HepPar-1) revealed that they were positive for HepPar-1, and some cells with biliary features were positive for CK7. Therefore, the histopathologic findings were suggestive of cHCC-CC (Figure 2). Intraoperatively, the mass was found to perforate the visceral peritoneum with no obvious regional lymph node or distant metastasis, and therefore, stage IIIA cHCC-CC was diagnosed. The tumor-node-metastasis staging criteria for cHCC-CC have been described in the 8^th^ edition of the American Joint Committee on Cancer/Union for International Cancer Control (11).

**Figure 3:**
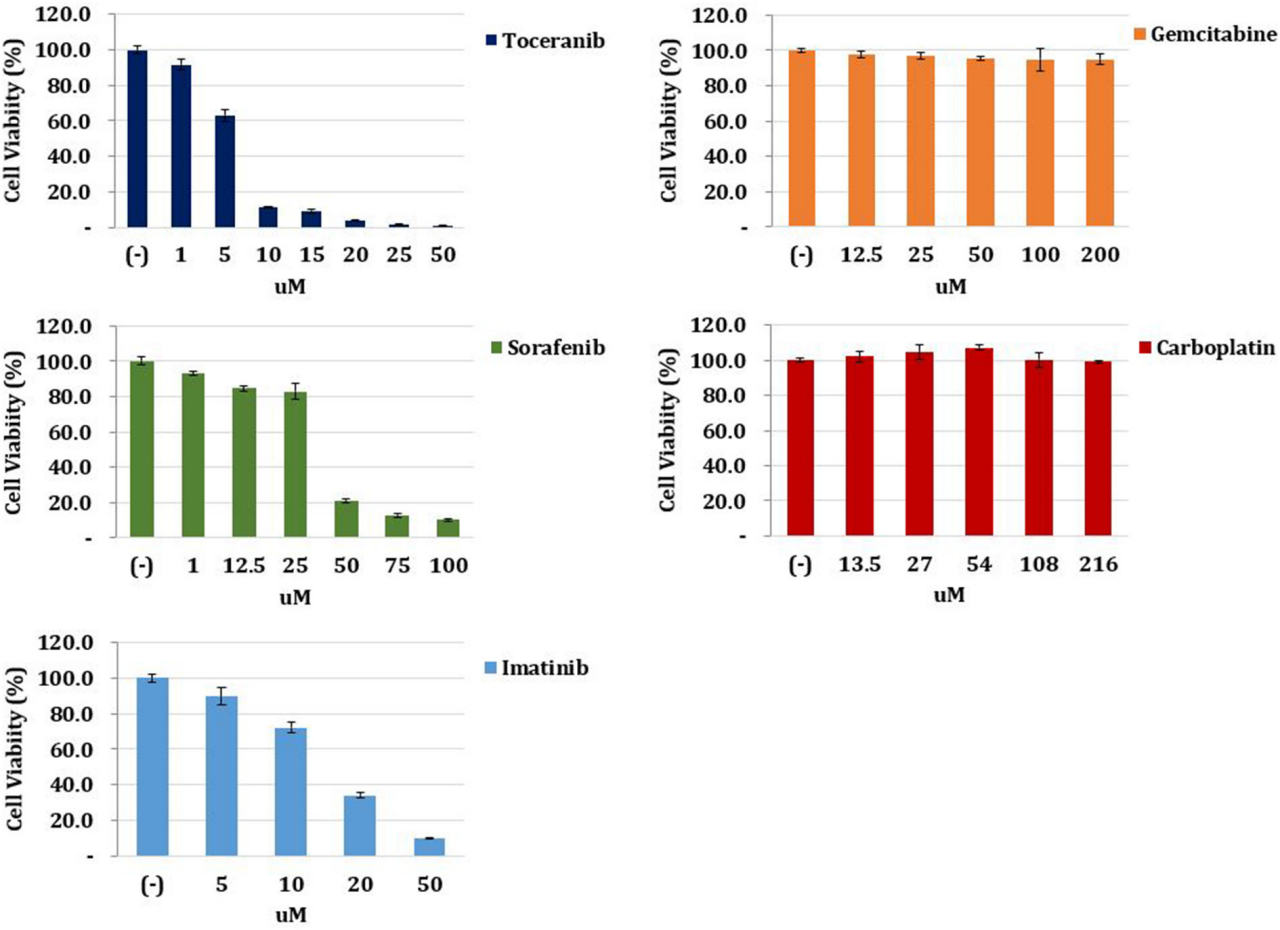
Anticancer drug response prediction test. The tumor tissue obtained from the patient was harvested, lysed, and filtered. The tumor sample was dissociated into single cells and cultured in medium. Cells were seeded and incubated in 96-well plates, and the medium was replaced with that containing gradient doses of chemotherapy drugs. After 24 h, cell viability was examined. The result revealed that toceranib had the best cancer cell-killing effect among gemcitabine, carboplatin, toceranib, sorafenib, and imatinib.

To select the most effective anticancer drug for this patient, we conducted an ATP-based anticancer drug response prediction test using a cell viability assay. After surgery, a tumor sample was harvested and lysed by collagenase II (Life Technologies, Carlsbad, CA, USA), hyaluronidase, and Ly27632 (Sigma-Aldrich, St. Louis, MO, USA) at 37°C. After filtering through a 70-μm cell strainer (BD Biosciences, San Diego, CA, USA), the tumor sample was dissociated into single cells and cultured in Advanced Dulbecco's modified Eagle's medium/F12 supplemented with 10% fetal bovine serum, 10 mM N-2-hydroxyethylpiperazine-N'-2-ethanesulfonic acid, and GlutaMAX™ (Thermo Fisher Scientific, Waltham, MA, USA), and Zellshield® (Minerva Biolabs, Berlin, Germany). Cells (1 × 10^4^) were seeded in 96-well plates and incubated overnight at 37°C in a humidified atmosphere of 5% CO_2_. The medium was replaced with that containing gradient doses of chemotherapy drugs. After 24 h, cell viability was examined using the CellTiter-Glo® Luminescent Cell Viability Assay (Promega, Madison, WI, USA). The result revealed that toceranib (Palladia®, Zoetis, USA) had the best cancer cell-killing effect among gemcitabine, carboplatin, toceranib, sorafenib, and imatinib (Figure 3). Therefore, it was elected to start toceranib as an adjuvant chemotherapeutic agent (3.1 mg/kg, PO, q48 h). We also prescribed trilostane (Vetoryl®, Dechra, UK) for managing hyperadrenocorticism at an initial dose (1 mg/kg, PO, q12 h) and final dose (5 mg/kg, PO, q12 h). The dosage was adjusted according to ACTH stimulation test results, and the patient did not show side effects of trilostane, such as lethargy and vomiting.

**Figure 4:**
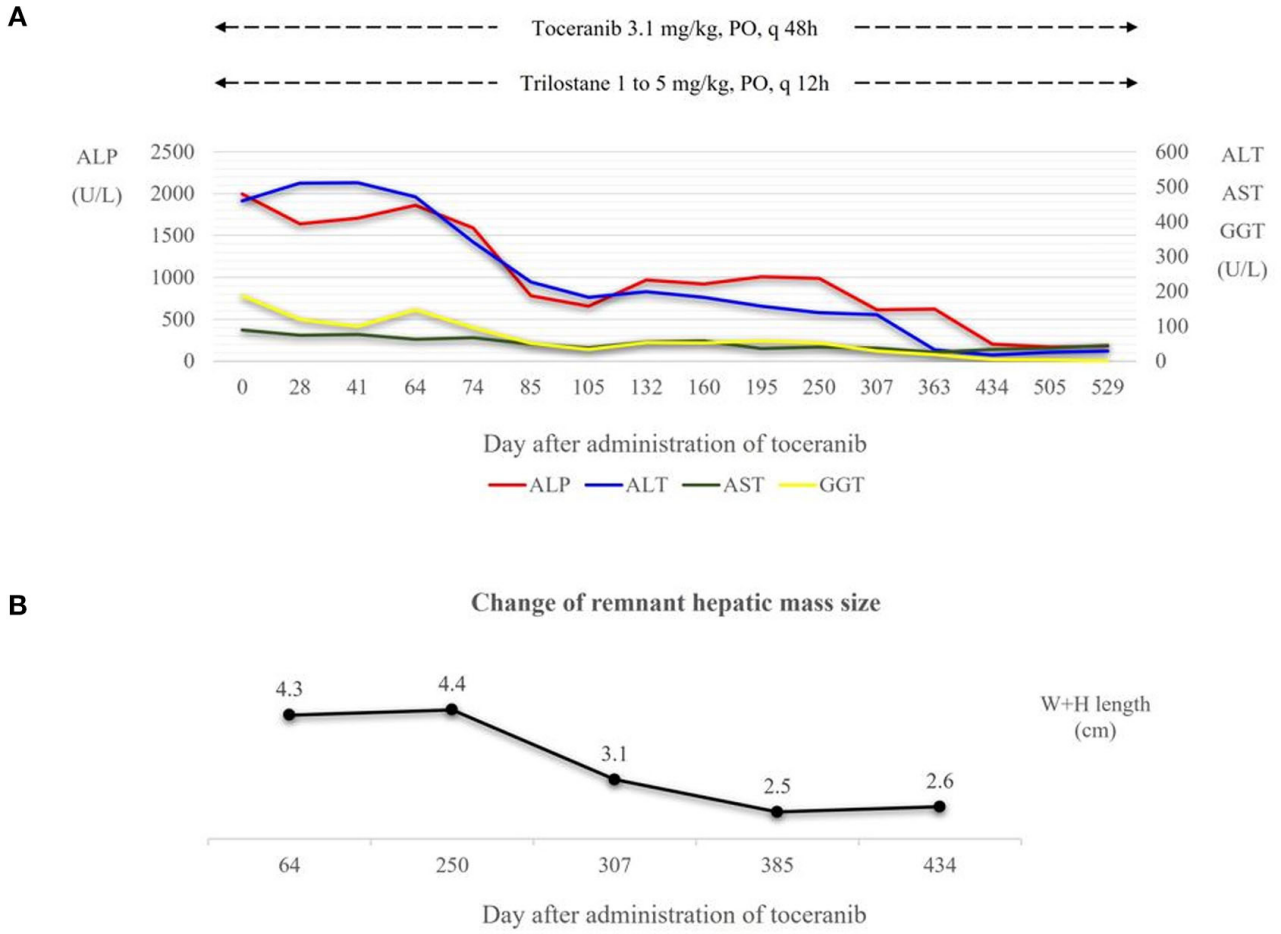
**(A)** Dose of toceranib and trilostane administered and hepatic enzyme level following the administration of toceranib. Trilostane was prescribed at initial presentation and dose escalation was conducted according to the ACTH stimulation test results and clinical signs of hyperadrenocorticism. At toceranib day 74, trilostane was escalated to the final dose (5 mg/kg, PO, q12h). Hepatic enzyme levels decreased gradually after the administration of toceranib, and the levels of all four hepatic enzymes appeared within the normal range since toceranib day 434. **(B)** Change in the remnant hepatic mass size in terms of the sum of its width and height measured on abdominal ultrasonography. The mass size was measured to 2.3 × 2.0 cm on toceranib day 64, 1.7 × 1.4 cm on toceranib day 307, and 1.6 × 1.0 cm on toceranib day 434. ALP, Alkaline Phosphatase. ALT, Alanine Aminotransferase. AST, Aspartate Aminotransferase. GGT, Gamma Glutamyltransferase. W+H length, width plus height length.

Recheck evaluations were completed every 2 weeks for the first 2 months, and then every 2 months subsequently for a routine check-up including systemic blood pressure measurement, CBC, serum chemistry profile, coagulation profile and urinalysis. Radiography and ultrasonography were conducted at one-month intervals for the first 2 months and every 2 months thereafter. The hepatic enzyme levels decreased gradually and have been within the normal range since toceranib day 434 (Figure 4A). A second CT scan performed on toceranib day 113 revealed a remnant hepatic mass measuring 3.3 × 2.7 × 2.1 cm but showed no evidence of metastasis. On abdominal ultrasonography, the remnant mass size reduced gradually and was 1.6 × 1.0 cm at toceranib day 434 (Figure 4B). Serial monitoring of the coagulation profile revealed a persistently normal D-dimer level and elevated MA on TEG.

However, urinalysis and serum chemistry profile showed apparently elevated UPC (9.78; reference range, < 0.5) and decreased serum albumin (2.1 mg/dL; reference range, 2.2–3.9 mg/dL) levels on toceranib day 251. At this point, the systemic blood pressure was within the normal range, so the renal loss of albumin was thought to be due to local glomerular hypertension. Ramipril (Vasotop®, MSD Animal Health, USA) was started to reduce the renal loss of albumin (0.125 mg/kg, PO, q24 h). Thereafter, UPC values decreased gradually to 2.12, and the serum albumin level has remained at the lower limit of normal range.

The patient is still alive over 23 months after partial hepatectomy. Side effects such as neutropenia, hypertension, and gastrointestinal signs have not been observed since the initiation of toceranib.

## Discussion

The clinical signs of hepatobiliary tumors are vague and nonspecific even in late stages, and most commonly include lethargy, anorexia, vomiting, and weight loss (1, 4, 12). Other clinical signs, such as polyuria, polydipsia, diarrhea, jaundice, dyspnea, seizure, hematochezia, and melena, have been reported but are less common. In one study, five of the 18 dogs did not show any clinical signs at the time of diagnosis (1). Therefore, detection and diagnosis of hepatobiliary tumors are challenging (12).

The patient reported here was presented for investigation of elevated hepatic enzymes, and the clinical signs reported by the owner were panting and polyphagia. The patient had no history of gastrointestinal signs, such as anorexia, vomiting, and diarrhea, and these clinical signs were attributed to hyperadrenocorticism. Even though the hepatic mass was diagnosed as stage IIIA cHCC-CC because of visceral peritoneum perforation and remarkable tumor adhesion to the diaphragm, the patient did not show any clinical signs caused by the tumor. This characteristic of the hepatobiliary tumor contributed to the late presentation of the patient to our hospital and, consequently, the poorer prognosis.

Dogs with hepatobiliary tumors may show prolonged coagulation times because most of the factors included in the coagulation process are produced by the liver (2, 13). In this case, the coagulation profile revealed delayed aPTT while the R time, K time, and alpha angle on TEG were within the normal range. However, elevation of MA was observed, and therefore, the test results were conflicting. TEG is a reliable assay to evaluate the kinetics of clot formation as a dynamic process (13). Medical treatment was not required because serial monitoring of TEG did not reveal a hypocoagulable state.

The prognosis for canine cHCC-CC is different from that of HCC or CC. The prognosis for canine HCC depends mainly on its morphology and feasibility of surgical excision (14). Among the three morphologic types, massive HCC is potentially resectable, and the median survival time is >1,460 days and 270 days in dogs that receive and cannot receive surgery, respectively (15). On the other hand, CC is a more malignant tumor compared to HCC, and a poor prognosis has been reported in both humans and dogs (16). The median survival time for dogs with CC is reported to be <6 months due to local recurrence and metastasis (2, 4, 12). A previous study reported a median survival time of 700 days in 14 dogs with massive type cHCC-CC who underwent complete surgical removal (17). However, research on the prognosis of incompletely resected or unresectable canine cHCC-CC is lacking.

Considering that partial tumor tissue remained unresected in this patient, adjuvant chemotherapy was highly recommended. However, there are no published reports describing antitumor agents for canine cHCC-CC because of its extremely low incidence. Similarly, limited evidence on systemic chemotherapy in human cHCC-CC leads clinicians to rely on case reports and small retrospective studies (18). Treatment decisions of human cHCC-CC are also extrapolated from those for HCC and CC (18).

In human medicine, sorafenib has been the gold standard first-line systemic treatment for advanced HCC (19). On the other hand, gemcitabine and platinum-containing chemotherapy regimens have been identified to show superior response rates and tumor control rates in CC (20). Additionally, imatinib mesylate has been shown to induce apoptosis in human CC cells (21).

The most currently approved first- and second-line treatments for human HCC target angiogenic pathways (22). Among several angiogenic pathways, the vascular endothelial growth factor (VEGF)/VEGFR signaling pathway has been validated as a drug target in HCC (22). Also, in one human study (23), growth factor analysis of CC samples from 41 patients showed that PDGF-BB and connective tissue growth factor were the most abundant growth factors, so targeting PDGFR-β may sensitize CC cells to apoptotic stimulation and have therapeutic effects *in vivo*. In another study on a CC rat model (24), stem cell factor (SCF) mRNA and c-KIT mRNA were upregulated up to 20-fold and 5-fold, respectively, in CC tissue in comparison with normal liver tissue. Thus, the SCF-c-KIT system may contribute to the development and proliferation of CC cells, and targeting this axis may have advantageous clinical effects.

For the anticancer drug response prediction test, we chose gemcitabine, carboplatin, sorafenib, and imatinib based on the abovementioned studies in human medicine. Toceranib was included because it is a multi-targeted tyrosine kinase inhibitor and the only FDA-approved agent for use in dogs (25). Furthermore, a previous study had reported on the chemotherapeutic effects of toceranib in unresectable massive type canine HCC (14). On the anticancer drug response prediction test, toceranib showed the best cancer cell-killing effect among five agents, including conventional and targeted chemotherapy agents. Therefore, the administration of toceranib was thought to be the best choice in this patient. Previous studies provided a basis to understand the optimal effects of toceranib in this patient.

The size of the remnant liver mass in this patient decreased gradually since the initiation of toceranib, and the sum of the diameters of the remnant mass reduced by more than 30%. Therefore, we assessed the response to toceranib as a partial response (PR) according to the response evaluation criteria for solid tumors in dogs of a Veterinary Cooperative Oncology Group (26).

The reported adverse effects of toceranib include anorexia, vomiting, diarrhea, neutropenia, hypertension, and proteinuria; therefore, serial monitoring of CBC, systolic blood pressure, and UPC are recommended for patients receiving this drug (27, 28). Our patient did not show hematologic toxicities, such as neutropenia, or gastrointestinal toxicities, such as vomiting and diarrhea. The patient's systolic blood pressure was consistently within normal range, but UPC was elevated significantly 8 months after administering toceranib. However, the patient already showed proteinuria even before taking toceranib, and hyperadrenocorticism was thought to be the most likely cause of proteinuria. At the time when the patient's proteinuria was exacerbated, she had been receiving trilostane at 5 mg/kg twice a day, but her post-ACTH cortisol was over 10 μg/dL, indicating insufficient suppression. We were concerned about the side effects of trilostane at doses over 5 mg/kg, hence, dose escalation was discontinued. Therefore, exacerbation of proteinuria was thought to be caused by insufficient suppression of cortisol rather than due to administration of toceranib.

This case study has some limitations. First, we could not perform accurate size monitoring of the remnant mass by serial CT scans. At the request of the owner, serial size monitoring was performed by ultrasonography due to the patient's high risk for repeated anesthesia, considering old age and the hepatobiliary tumor. Second, we could not compare the RTK gene expression of the tumor tissue and normal hepatobiliary tissue from this patient. Nevertheless, the size of the remnant tumor reduced after administration of toceranib, suggesting that toceranib had a therapeutic effect on the tumor in this patient. Finally, this is a single patient case report, so the effect of toceranib on other dogs with cHCC-CC cannot be predicted.

In conclusion, this is the first case report to describe chemotherapy in a dog with cHCC-CC. Toceranib seemed effective, and the patient is still alive over 23 months after tumor excision. The patient had not shown any critical adverse effects of toceranib or obvious evidence of tumor recurrence or metastasis including at the most recent follow-up assessment. Additional studies assessing the effectiveness of tyrosine kinase inhibitors as an alternative to conventional chemotherapy for dogs with cHCC-CC are warranted.

## Data availability statement

The original contributions presented in the study are included in the article/supplementary material, further inquiries can be directed to the corresponding author.

## Ethics statement

Ethical review and approval was not required for the animal study because it was not required for case report. Written informed consent was obtained from the owners for the participation of their animals in this study.

## Author contributions

S-WK wrote the article and contributed to clinical evaluation, diagnosis, treatment, and follow-up of the patient. J-WC and J-MK contributed to clinical evaluation and diagnostic work-up of the patient and helped write the manuscript. H-YY contributed to the surgery and post-operative management of the patient and helped write the manuscript. KB and K-AY contributed to the molecular work and helped write the manuscript. J-HK supervised the clinical evaluation, diagnosis, and treatment of the patient and wrote the manuscript. All authors read, revised, and approved the final manuscript.

## Funding

This work was supported by the National Research Foundation of Korea Grant funded by the Korean Government (MSIT)(NRF-2021R1A2C2008112).

## Conflict of interest

The authors declare that the research was conducted in the absence of any commercial or financial relationships that could be construed as a potential conflict of interest.

## Publisher's note

All claims expressed in this article are solely those of the authors and do not necessarily represent those of their affiliated organizations, or those of the publisher, the editors and the reviewers. Any product that may be evaluated in this article, or claim that may be made by its manufacturer, is not guaranteed or endorsed by the publisher.
